# Investigation of individual cultural values and perceived gender role on disaster management in Generation Z earthquake victims

**DOI:** 10.3389/fpsyg.2025.1671334

**Published:** 2025-11-19

**Authors:** Ibrahim Akkaş, Semra Bulbuloglu, Sinan Aslan, Ahmet Cetintas

**Affiliations:** 1 Department of Social Service Policies and Practices, Faculty of Economics and Administrative Sciences, Erzincan Binali Yildirim University, Erzincan, Türkiye; 2 Division of Surgical Nursing, Department of Nursing, Faculty of Health Sciences, Istanbul Aydin University, Istanbul, Türkiye; 3 Department of Midwifery, Faculty of Health Sciences, Istanbul Aydin University, Istanbul, Türkiye; 4 Department of Nursing Faculty of Health Sciences, Kahramanmaras Istiklal University, Kahramanmaras, Türkiye; 5 Department of Social Work, Faculty of Health Sciences, Malatya Turgut Ozal University, Malatya, Türkiye

**Keywords:** disaster management, earthquake, earthquake victims, Generation Z, individual cultural values, perceived gender role

## Abstract

**Introduction:**

High magnitude earthquakes are a natural disaster, often resulting in psychosocial and economic devastation. In patriarchal societies, women may suffer more losses than men and may be disadvantaged in earthquake management. Generation Z has more independent personal characteristics than previous generations, which may have an impact on gender perceptions and individual cultural values on earthquake management.

**Objective:**

This study aimed to examine the gender perception and individual cultural values of Generation Z earthquake victims on earthquake management.

**Method:**

This descriptive study was conducted in Malatya, one of the cities affected by the earthquake on February 6, 2023, 2 years after the earthquake, with the participation of *n* = 226 Generation Z earthquake victims who married or sharing a home with a partner for at least 6 months. Earthquake victim information form, Perception of Gender Role on Disaster Management Scale (PGR-DMS) and Individual Culture Values Scale (CVSCALE) were used for data collection. The PGR-DMS measures gender perception, and higher scores indicate a more positive gender perception. CVSCALE measures individual cultural values and has five sub-dimensions: Power Distance, Uncertainty Avoidance, Collectivism, Long-Term Orientation and Masculinity. Higher score on a subscale indicates that participants embrace the cultural value measured in that dimension for CVSCALE. Descriptive tests, Mann–Whitney U, Chi Squared and Spearman Rho correlation tests were used in data analysis.

**Results:**

75.7% of the Generation Z earthquake victims who participated in the study were between 21 and 25 years old and 72.6% were female. 63.3% of Generation Z earthquake victims were high school graduates and 74.3% were employed. 20.8% of Generation Z earthquake victims had a chronic disease, and 15.5% of them had a spouse with a chronic disease. As a result of this study, the PGR-DMS was higher in earthquake victims whose spouses had chronic diseases and this difference was statistically significant (*p* = 0.030). There was a statistically significant strong relationship between the number of children and PGR-DMS (*r* = 0.711, *p* = 0.014). There was a positive, strong and statistically significant correlation between PGR-DMS and Collectivism (*r* = 0.754, *p* = 0.021).

**Conclusion:**

As a result of this study, having a chronic disease in the spouse of Generation Z earthquake victims and an increase in the number of children positively increased the perception of gender in earthquake management. Generation Z earthquake victims’ perception of gender in earthquake management, Collectivism and Long-term orientation were above average. As the collectivism of Generation Z earthquake victims increased, the perception of gender in earthquake management raised positively.

## Introduction

On February 6, 2023, two separate earthquakes of magnitude 7.7 and 7.6 Magnitude (Mw) occurred approximately 8 h apart in Kahramanmaraş, a large city in eastern Turkey. These earthquakes were reported to have repeatedly ruptured adjacent active fault systems from Hatay to Malatya in Turkey (Karabacak et al., 2023) and were recorded in world history as “the disaster of the century” (Kanwal, 2023). In recent decades, there have been numerous natural disasters such as earthquakes, landslides, droughts, floods, tropical storms, forest fires and volcanic eruptions (Valdés, 2019). Natural disasters are unpredictable when and where they occur, and civil society’s protective mechanisms often fail (McFarlane and Williams, 2012; Usta et al., 2024). High magnitude earthquakes and other disasters cause societies to be deeply shaken by their severity. Reactions to the destruction caused by natural disasters vary according to the cultural, social, economic and political characteristics of individuals (Mehta, 2007; Wei et al., 2020).

Although the majority of individuals who experience traumatic events due to natural disasters do not develop psychopathology, natural disasters can threaten our psychological well-being in many ways, causing short and long-term psychological distress and various psychological symptoms (Saeed and Gargano, 2022; Lee et al., 2015). It has been reported that men and women react differently to natural disasters in various parts of the world and their coping strategies vary (Enarson and Meyreles, 2004). In this context, it has been reported that women belonging to different social classes, races, ethnic and age groups are more vulnerable than men belonging to the same social class/group before, during and after disasters (Ariyabandu, 2019). This difference between the two genders may stem from biological and physiological dynamics. In addition, social norms and role behaviors may cause women to exhibit behaviors that increase their vulnerability during disasters. Apart from all these, disasters may lead to scarcity of basic resources, unequal access to resources and temporary disruption of social order. Difficulties in accessing basic necessities exacerbate competition among individuals, leading to social inequality, disadvantaging the female gender and paving the way for the emergence of new forms of discrimination (Brown, 2000; Bradley et al., 2022; Blaikie et al., 1994; UN Women, 2023).

Women are estimated to be more vulnerable than men during disasters, and this vulnerability stems as much from the general position of women in society as from the disaster itself (Neumayer and Plümper, 2007; Reyes and Lu, 2016). Gender inequality increases the social burden of disasters. Women’s submission to the patriarchal structure is recognized as the main reason for disaster vulnerability (Enarson, 2000). Until now, individual cultural values and gender perception have had a high impact on individuals’ responses to social disasters. However, this situation may be different for Generation Z, which was born during the period of increased internet use.

Generation Z is the first digital native Generation born in the world of internet-connected technology. However, there is no specific definition of the birth time of Generation Z members because they were born and raised in a digital and technological environment, learned how to use technology and interacted in social networks from a very young age. Some sociologists define them as those born after 1995, while others say they were born after 1997 (Szymkowiak et al., 2021; Lanier, 2017). Some sociologists argue that those born after 2000 can be considered as Generation Z (Poláková and Klímová, 2019; Snieska et al., 2020). Unlike their predecessors, Generation Z did not grow up with extended family structures (e.g., single-parent, same-sex couples, multi-generational families) and evolving gender norms. The consequences of Generation Z growing up more freely are reflected in policy and brand preferences beyond gender and sexuality (Stahl and Literat, 2023). In this sense, it is thought that individuals belonging to Generation Z have a variable structure in terms of gender perception and are far from adopting traditional gender roles. In this context, the individual cultural values of Generation Z differ significantly from previous generations, and their perceived gender roles on disaster management are also thought to differ. No studies have been found in the literature examining the individual cultural values and perceived gender roles on disaster management of Generation Z earthquake victims. Individual cultural values encompass several components, including an individual’s perspective on equality and power, their relationship with the unknown, and their relationships with other people. This study’s research questions were based on the relationship between individual cultural values and perceived gender roles in disaster management, and that this relationship may take on a different dimension in Generation Z earthquake victims. In this study, we aimed to examine the gender perception and individual cultural values of Generation Z earthquake victims on earthquake management.

## Materials and methods

This study was conducted 2 years after the Kahramanmaras-centered earthquake to investigate the Individual Cultural Values and Perceived Gender Role on Disaster Management in Generation Z earthquake victims.

### Research design and participants

This study is descriptive and cross-sectional. One of the cities affected by the Kahramanmaras-centered earthquake is Malatya in eastern Turkey. It was conducted with the participation of Generation Z victims who experienced the earthquake in Malatya eastern Turkey and are still living in that region. Data collection was done in container sites in Malatya. The sample was selected using a purposive sampling method. G*Power-3.1.9.2 software was used to calculate the sample size. When calculating the sample size, at least *n* = 104 earthquake victims were required to participate in the study with 0.05 margin of error, 0.4 effect size, and 95% confidence interval. The sample of this study consisted of *n* = 226 earthquake victims. Generation Z earthquake victims from whom written informed consents were taken filled in the questionnaires. The inclusion and exclusion criteria for earthquake victims are given below.

### Inclusion and exclusion criteria

The inclusion criteria for this study were (i) Being in the earthquake zone during the Malatya earthquake; (ii) Being over 18 years of age and in Generation Z, not having any communication, language or psychiatric problems; (iii) Volunteering to participate in the study; (iv) Continuing to live in the same region after the earthquake; (v) Having a continuing relationship for at least 6 months, sharing the same house with her/his spouse and partner for at least 6 months. The opposite of these criteria was determined as exclusion criteria.

### Data collection and tools

Generation Z earthquake victims visited by researchers in container cities. Data collection forms were transferred to the online environment by researchers. After reading the informed consent text, Generation Z earthquake victims approved to participate in the study and proceeded to the questions. Earthquake victim information form, Perception of Gender Role on Disaster Management Scale, Individual Culture Values Scale were used for data collection. Information about the data collection tools is presented below.

### Earthquake victim information form

The earthquake victim information form is a questionnaire that includes questions which age, gender, marital status, education status, economic status, previous living site, relationship duration, family type, job status, spouse’s employment status, chronic diseases of the spouse and self, and number of children.

### Perception of gender role on disaster management scale

The Perception of Gender Role on Disaster Management Scale (PGR-DMS) was developed by Onal et al. (2022). The internal consistency of PGR-DMS was determined as 0.77 Cronbach’s alpha value (Onal et al., 2022). The scale has no subdimensions. The scale consists of 19 items. Two of these items are positive and 17 are negative. Negative items were recoded before analysis. A total score between 19 and 95 is obtained from the scale. As the total score obtained from the scale increases, negative perception decreases. Accordingly, 19–34 points represent full negative perception, 35–49 points represent negative perception, 50–64 points represent neutral perception, 65–79 points represent positive perception, and 80–95 points represent full positive perception. The Cronbach’s alpha value for this study was 0.71.

### Individual culture values scale

The Individual Culture Values Scale (CVSCALE) was developed by Hofstede (1980). CVSCALE is Hofstede’s five-dimensional scale at the societal level and was created by considering it in an individual context (Hofstede, 1980, 2001, 2011). The Turkish validity and reliability analyses of the scale were conducted by Saylık (2019). The Likert-type scale, which deals with Hofstede’s (1980) socio-cultural values, consists of 26 items and five subscales. Among the subscales, power distance and uncertainty avoidance are explained with 5 items each, collectivism and long-term orientation with 6 items each, and masculinity with 4 items. The power distance sub-dimension considers the extent to which inequality and power are appropriate. Uncertainty avoidance sub-dimension considers how unknown situations, uncertainty, and unexpected events are handled. Collectivism sub-dimension considers the extent to which individuals are integrated into groups and their perceived obligations and dependence on them. The long-term orientation sub-dimension addresses society’s perspective on the time horizon. The masculinity sub-dimension examines the value society places on traditional male and female roles. A total score cannot be obtained from the CVSCALE. The score from each sub-dimension is divided by the number of items in that dimension and converted to a mean. Higher sub-dimension scores indicate greater belief in the cultural values in that sub-dimension. Saylık (2019) determined the Cronbach’s Alpha reliability coefficient of the entire scale as 0.80. Cronbach’s alpha value for this study was 0.76.

### Statistical analysis

Statistical Package for the Social Sciences (SPSS) 27.0 IBM (Armonk, NY, USA) was used to analyze the data of this study. Firstly, it was determined that they did not show normal distribution with the Kolmogorov–Smirnov test. Descriptive statistical methods (frequency, percentage, mean, standard deviation, minimum and maximum value) were used to calculate numbers, percentages and means. Mann–Whitney U and Chi-squared tests were used to compare independent groups. The Spearman rho correlation test was performed between the PGR-DMS and CVSCALE subscales. Furthermore, the Spearman rho correlation test was performed between the number of children and age variables and the PGR-DMS. The numerical results obtained were evaluated at *p* < 0.05 statistical significance level and a 95% confidence interval.

### Ethical aspects of the study

This research was approved by Erzincan Binali Yıldırım University Social and Human Sciences Research Ethics Committee (Date: 27 Feb 2025, Number: 02/02). Informed consent was obtained from each earthquake victim in line with the Declaration of Helsinki. Participants who gave consent on the online platform then proceeded to the questionnaires.

## Findings

Table 1 shows the individual characteristics of Generation Z earthquake victims, PGR-DMS and statistical tests. Of the earthquake victims who participated in the study, 75.7% were between the ages of 21 and 25, 72.6% were female and married. 63.3% of the earthquake victims were high school graduates, 61.9% had middle income, 64.2% lived in a town in previously, 54% had been married between 6 and 10 years, 75.7% had a large family, 74.3% had a job, and 67.3% had a spouse with a job. 20.8% of the earthquake victims had a chronic disease, and 15.5% of their spouses had a chronic disease. Individual characteristics did not affect gender perception in earthquake management. However, earthquake victims whose spouses had chronic diseases had a more positive perception of gender in earthquake management and this difference was statistically significant (*p* = 0.030). There was a positive, strong and statistically significant relationship between the number of children and the perception of gender in earthquake management (*r* = 0.711, *p* = 0.014). In this context, as the number of children increased, earthquake victims had a more positive perception of gender in earthquake management.

**Table 1:** Individual characteristics, PGR-DMS scores of Generation Z earthquake victims, and comparisons.

Characteristics		*n* (%)	PGR-DMS	Comparison of PGR-DMS and characteristics
			Mean ± Sd	
Age	18–20	55 (24.3)	67.98 ± 8.42	*U* = 4,479, *p* = 0.596
	21–25	171 (75.7)	68.59 ± 9.46	
Gender	Female	164 (72.6)	67.92 ± 9.16	*U* = 4,474, *p* = 0.164
	Male	62 (27.4)	69.80 ± 9.24	
Relationship	Married	164 (72.6)	67.92 ± 9.16	*U* = 4,414, *p* = 0.101
	Partnered	62 (27.4)	69.80 ± 9.24	
Education	Primary	49 (21.7)	68.46 ± 8.17	*χ*^2^ = 1,063, *p* = 0.786
	High school	143 (63.3)	68.13 ± 9.59	
	Associate’s	17 (7.5)	70.76 ± 8.11	
	BSc and above	17 (7.5)	68.64 ± 10.09	
Economic	Low	62 (27.4)	68.56 ± 8.37	*χ*^2^ = 0.071, *p* = 0.965
	Middle	140 (61.9)	68.25 ± 9.52	
	High	24 (10.6)	69.25 ± 9.66	
Previous living site	City center	21 (9.3)	65.47 ± 7.54	*χ*^2^ = 2,943, *p* = 0.230
	District-town	145 (64.2)	68.73 ± 9.66	
	Village	60 (27.5)	68.76 ± 8.50	
Relationship duration (years)	5 or less	35 (15.5)	66.80 ± 7.92	χ^2^ = 1,960, *p* = 0.581
	6 to 10	122 (54)	68.73 ± 9.78	
	11 to 15	31 (13.7)	69.19 ± 8.63	
	16 and above	38 (16.8)	68.31 ± 9.00	
Family type	Nuclear	55 (24.3)	67.98 ± 8.42	*U* = 4,479, *p* = 0.596
	Extended	171 (75.7)	68.59 ± 9.46	
Job status	Employed	168 (74.3)	68.19 ± 9.25	*U* = 4,570, *p* = 0.481
	Unemployed	58 (25.7)	69.15 ± 9.08	
Spouse’s employment	Employed	152 (67.3)	68.32 ± 9.12	*U* = 5,436, *p* = 0.683
	Unemployed	74 (32.7)	68.68 ± 9.43	
Chronic disease	No	179 (79.2)	68.05 ± 9.15	*U* = 3,614, *p* = 0.138
	Yes	47 (20.8)	69.91 ± 9.33	
Spouse’s chronic disease	No	191 (84.5)	65.45 ± 9.16	*U* = 2,573, ***p* = 0.030**
	Yes	35 (15.5)	68.98 ± 9.13	
Number of children (Mean ± Sd)		1.87 ± 0.66 (Min 1, Max 4)		*r* = 0.711, ***p* = 0.014**

*χ*^2^, Chi Squared test; *U*, Mann Whitney U test, *r*, Spearman’s rho correlation test, *p < 0.05, **p < 0.01.

Table 2 shows the PGR-DMS and CVSCALE scores of the Generation Z earthquake victims. The total scores obtained from PGR-DMS was 68.44 ± 9.20. When the scores of CVSCALE sub-dimensions were examined that they were Power Distance, Uncertainty Avoidance, Collectivism, Long-Term Orientation and Masculinity, respectively, 2.41 ± 0.83, 3.64 ± 1.01, 4.76 ± 0.39, 4.26 ± 0.39, and 3.75 ± 0.61.

**Table 2:** PGR-DMS and CVSCALE scores of Generation Z earthquake victims.

Total and subdimension scores of the scales	Items	Score range	x̄ ± Sd	Min–Max
PGR-DMS	1–19	19–95	68.44 ± 9.20	41–89
CVSCALE*	–	–	–	–
Power distance	1–5	1–5	2.41 ± 0.83	1–5
Uncertainty avoidance	6–10	1–5	3.64 ± 1.01	1–5
Collectivism	11–16	1–6	4.76 ± 0.39	1–6
Long-term orientation	17–22	1–6	4.26 ± 0.39	1–5
Masculinity	23–26	1–4	3.75 ± 0.61	1–4

*Total score is not taken into account.

Table 3 shows the correlation analysis between mean scores of PGR-DMS and CVSCALE sub-dimensions of Generation Z earthquake victims. There was a weak and statistically significant negative correlation between PGR-DMS and CVSCALE sub-dimensions of Power Distance (*r* = −0.248, *p* = 0.000), Uncertainty Avoidance (*r* = −0.166, *p* = 0.013) and Masculinity (*r* = −0.212, *p* = 0.001). There was a positive, strong and statistically significant correlation between PGR-DMS and Collectivism, one of the sub-dimensions of CVSCALE (*r* = 0.754, *p* = 0.021).

**Table 3:** Correlation analysis between scores of PGR-DMS and CVSCALE sub-dimensions of Generation Z earthquake victims.

Total and subdimension scores of the scales	PGR-DMS	
	*r*	*p*
CVSCALE	–	**–**
Power distance	−0.248	0.000**
Uncertainty avoidance	−0.166	0.013*
Collectivism	0.754	0.021*
Long-term orientation	0.010	0.878
Masculinity	−0.212	0.001**

*Correlation is significant at the 0.05 level (2-tailed).

**Correlation is significant at the 0.01 level (2-tailed).

*r*, Spearman’s rho correlation test.

**Figure 1:**
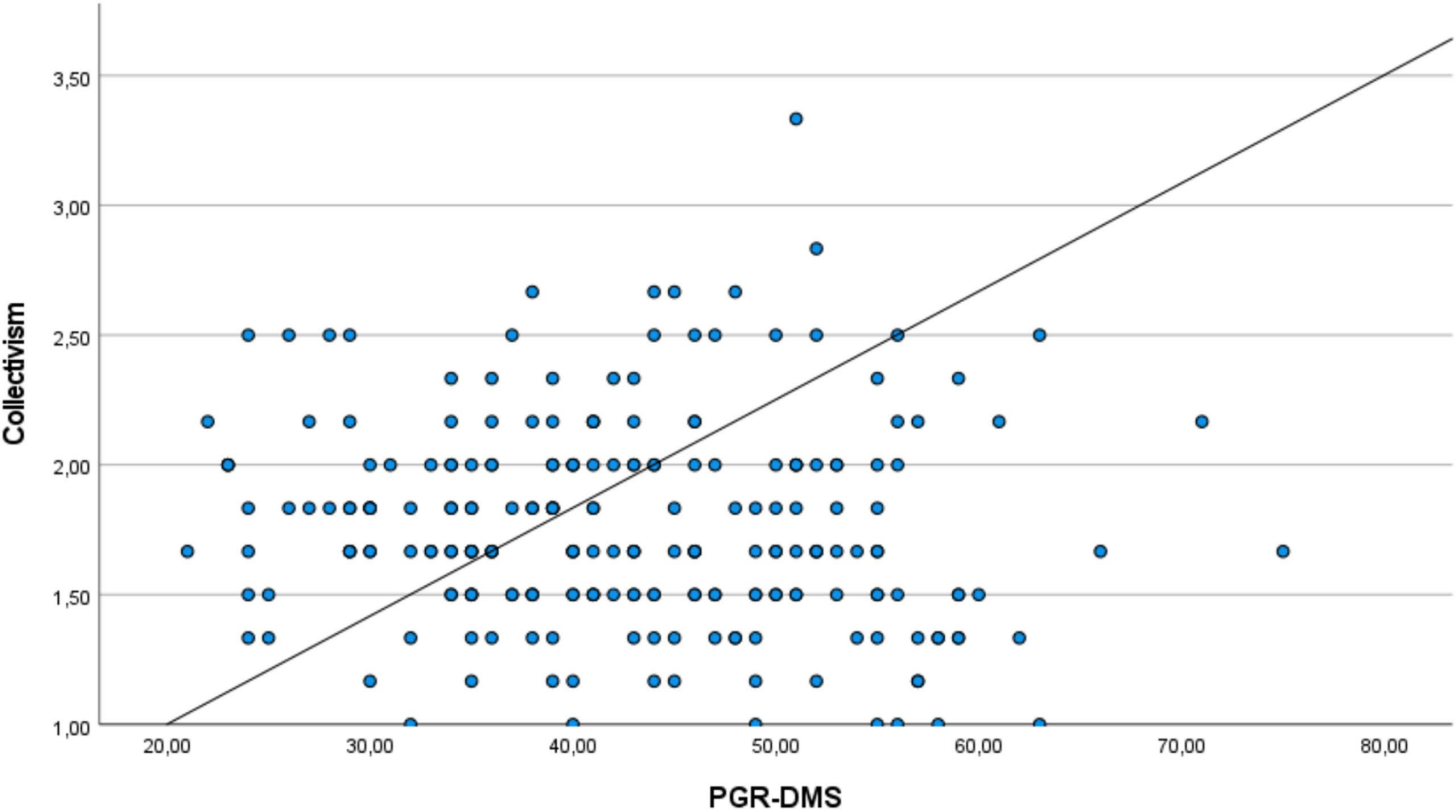
Comparison of PGR-DMS and collectivism scores of Generation Z earthquake victims.

Figure 1 shows the comparison of PGR-DMS and Collectivism mean scores of Generation Z earthquake victims. The same directional relationship between PGR-DMS and Collectivism is seen.

## Discussion

In this study, the relationship between Generation Z earthquake victims’ perceptions of gender in earthquake management and their individual cultural values was examined. The findings revealed that individuals with chronic illnesses in their spouses had more positive gender perceptions. Similarly, it was noted that the increase in the number of children positively affected gender perception. In previous studies, it has been stated that increased care responsibility leads to a more egalitarian perception of social roles (Connell, 2005; Hochschild and Machung, 2012). As a result of this study, we found that individual characteristics do not affect gender perceptions in earthquake management. The perceptions and actions of men and women during an earthquake are affected by the psychosocial destruction caused by the earthquake. Women and men share the common behaviors of experiencing the fear of facing the danger inherent in a disaster and taking various actions to save their families and relatives. In a study found that members of Generation Z struggle against climate change and report experiencing eco-anxiety, suffering and worrying about the natural events they experience (Tsevreni et al., 2023). Flooding in 2007 and 2008 in the UK showed that the gendered experiences of homes and communities are different for men and women. This suggests that people are unaware of existing gender inequality, as is the case with people in Kathmandu city and Sankhu town (Akerkar and Fordham, 2017; Thapa and Pathranarakul, 2019).

As a result of this study, no significant statistical relationship was found between age, gender, education level, family type, economic and marital status, duration of marriage and employment status and gender perception. Previous studies have revealed that after the earthquake, victims experienced high rates of depression, hopelessness, anxiety, and helplessness in addition to experiencing economic problems, economic stress, and financial difficulties (Alcover et al., 2022; Canlı and Yılmaz, 2024; Tekin and Aydin, 2025). Gender perception is shaped more by social roles and experiences rather than individual factors (Inglehart and Norris, 2003; Ridgeway, 2011; Wier et al., 2024). Experiencing a natural disaster involves concrete consequences experienced by individuals with or without victimization, and gender roles may shape the natural disaster experience. This conclusion is in line with Hofstede’s (2001) cultural dimensions theory. Participant statements reflect the influence of dimensions such as power distance, individualism–collectivism and uncertainty avoidance on communication styles. For example, in cultures with high power distance, individuals behave more distantly and formally in communication with authority figures, which may create conflict in interactions with individuals from cultures with low power distance (Hofstede, 2001; Lajnef and Ellouz, 2024; Lin and Lou, 2024).

In this study, the negative correlation between PGR-DMS and CVSCALE sub-dimensions (Power distance, Uncertainty avoidance and Masculinity) show that as individual cultural values increase, gender perception becomes less positive. In this context, it is seen that the perception of gender equality weakens in individualistic and authority-based social structures (Hofstede, 2011; House et al., 2004; Slick and Hertz, 2024). In addition, individuals with high power distance carry biases about women’s leadership roles in disaster management. This can result in women facing unique vulnerabilities during and after earthquakes (Eagly and Karau, 2002; Rahman, 2013; Rubbia, 2022; Blanchard, 2024; Rahmani et al., 2024; Özer et al., 2025; Wollast et al., 2025).

One of the dramatic results of this study is the strong and positive correlation between the PGR-DMS and the Collectivism subscale of the CVSCALE. As collectivism values increase in individuals, the perception of gender in earthquake management becomes more positive. This situation reveals that individuals with collective values prioritize solidarity in disaster periods and exhibit more egalitarian attitudes towards gender roles (Abdalla et al., 2024; Abid et al., 2025; Triandis, 2001; Yuki et al., 2005). In collectivist cultures, individuals exhibit behaviors based on common goals and cooperation. Accordingly, a previous study reported that gender-based discrimination is less common during a crisis (Jetten et al., 2017). It is of great importance to develop gender-sensitive policies to increase collective values and facilitate crisis management during earthquakes (Enarson and Chakrabarti, 2009). Developing federal policy and response strategies is crucial to reducing the impact of disasters on multicultural societies (Akdemir, 2024).

Relationships between genders stem from norms and roles (Triandis, 2001; Hofstede, 2011; Slick and Hertz, 2024). In this study, it was reported that with the increase in individualistic cultural values, gender perception in earthquake management was negatively affected. From this point of view, an increase in individualism may result in a decrease in social solidarity and the abandonment of collective responsibility-taking behavior. It was reported more than 20 years ago that individuals belonging to Generation Z may experience conflict with traditional patterns of gender roles in earthquake management (Prensky, 2001). The results obtained in our study support previous research findings. The high level of individual cultural values may cause women to remain in the background in disaster management compared to men. In this context, it is important to support egalitarian approaches in groups where individualistic values are high (Inglehart and Norris, 2003; Kahan et al., 2011).

In this study, Generation Z earthquake victims’ gender perception and individual cultural values on earthquake management were above the medium level. This result, similar to previous studies, shows that Generation Z individuals are more aware of disaster processes (United Nations Office for Disaster Risk Reduction-UNDRR, 2022; UN Women, 2020; Wisner et al., 2012). Generation Z individuals had a positive perception of gender about their high collectivism values, which allowed them to differentiate themselves from their predecessors. Generation Z has created the impression that they are more technologically skilled and independent, and they have gained a sensitive nature in terms of social justice and equality (Twenge, 2017; Seemiller and Grace, 2019). This shows that Generation Z is more prone to inclusive, fair and collective solutions in high-risk situations such as earthquake management (Slick and Hertz, 2024). This study revealed that individual cultural values are effective on Generation Z’s earthquake management in the perspective of gender perception. In this context, the determining role of collectivism, which is a sub-dimension of cultural values, draws attention. There are several limitations of this study. The sample size of this study was not very large, and the sample consisted only of a single city (Malatya) and voluntary participants. Past crisis and disaster experiences of the individuals in the sample were not questioned. The data collection tools were based on self-report, and the results were limited to the responses given by the participants. The social support systems of the individuals in the sample, other than the nuclear family, were not questioned; their existing social support may have shaped their earthquake management systems. All these were accepted as a limitation principle.

## Conclusion

In this study, gender perception and individual cultural values in earthquake management in Generation Z earthquake victims were examined. In this study of the earthquake victims who participated 75.7% were between the ages of 21 and 25, 72.6% were female. According to the results obtained, Generation Z earthquake victims whose spouses had chronic diseases had more positive gender perceptions in earthquake management. In addition, as the number of children increased, the positivity of gender perception in earthquake management increased. Apart from these two individual variables, age, gender, educational status, family type, economic and marital status, marriage duration and employment status did not affect the perception of gender in earthquake management. In this study, as perception of gender in earthquake management increased, the sub-dimensions of individual cultural value—power distance, uncertainty avoidance, and masculinity—decreased. Furthermore, other sub-dimension of individual cultural value, collectivism, increased with positive perception of gender in earthquake management.

## Data Availability

The original contributions presented in the study are included in the article/supplementary material, further inquiries can be directed to the corresponding author.
